# Reversible cerebral vasoconstriction syndrome with cardiac involvement during treatment for iron deficiency anemia: caser report

**DOI:** 10.1186/s12883-021-02509-w

**Published:** 2021-12-10

**Authors:** Kenya Oguchi, Kazuhiro Fukushima, Akinori Nakamura, Yo-ichi Takei

**Affiliations:** Department of Neurology, National Hospital Organization Matsumoto Medical Center, 2-20-30 Muraimachi-minami, Matsumoto, 399-8701 Japan

**Keywords:** Thunderclap headache, Reversible cerebral vasospasm syndrome (RCVS), Anemia, Cardiac involvement, Calcium channel blockers

## Abstract

**Background:**

The diagnosis and therapy of reversible cerebral vasoconstriction syndrome (RCVS) tends to focus on neurological symptoms, but less attention has been paid the occurrence of extracerebral lesion such as the myocardium.

**Case presentation:**

A 40-year-old woman taking iron supplements for iron deficiency anemia due to menorrhagia had suffered from a thunderclap headache and seizure. Brain magnetic resonance imaging revealed high-intensity lesions bilaterally in the cerebellar and cerebral hemispheres. Her symptoms once subsided with steroids and anticonvulsant therapy; however, she experienced a severe headache again while bathing and was transferred to our hospital. Based on the clinical course and imaging data, she was diagnosed as having RCVS triggered by a rapid improvement of anemia. At the same time, she had cardiac involvement revealed by electro and echocardiographs despite without chest symptoms. After the administration of a calcium channel blocker and nitrite, her cerebral and cardiac involvements were rapidly improved.

**Conclusions:**

The case presented RCVS with transient myocardial damage. With RCVS, we should always pay attention to the complication of extracerebral lesions.

## Background

Reversible cerebral vasoconstriction syndrome (RCVS), which has been reported to occur more frequently in women aged 20–50 years, is a rare disease with an unknown pathophysiology. Clinically, RCVS presents with a thunderclap headache, neurological symptoms, and convulsions. Reversible cerebral vasospasm, transient ischemic attacks, cerebral infarction, and subarachnoid hemorrhage have been proposed as the diagnostic criteria for RCVS [1]. The triggers and background factors of this disease include the postpartum period, migraine, hypertension, abuse of drugs such as stimulants, cannabis, cocaine, and alpha sympathomimetics, use of serotonin agonists [2], rapid improvement of anemia [3], temperature differences such as bathing with too hot or cold water [4], infections such as COVID-19 [5], vasculitis [6], and collagen diseases such as systemic lupus erythematosus [7]. The pathological mechanism behind RCVS is considered to be sympathetic overactivity, or vascular endothelial damage and dysfunction of vascular smooth muscle due to oxidative stress, and dilation or spasm of cerebral surface small blood vessels which contribute to form middle blood vessels and main arteries [8].

To date, little attention has been paid to the involvement of extracerebral organs in patients with RCVS, but some studies have reported that cardiac ventricular abnormalities or coronary artery spasm may be a part of the RCVS spectrum [9, 10]. Thus, vasoconstriction may not be limited to the cerebral vasculature and may involve the extracerebral organs.

Here, we report the case of a middle-aged Japanese woman with a history of migraine and undergoing treatment for iron deficiency anemia, who developed RCVS with cardiac involvement.

## Case presentation

A 40-year-old woman had suffered from a severe headache early in the morning and visited a nearby neurosurgical hospital. During her consultation with a neurologist, a generalized convulsion occurred starting from her right upper limb. She was administered 5 mg of diazepam intravenously, and brain magnetic resonance imaging (MRI) revealed high-intensity lesions bilaterally in the cerebellar hemispheres, and near the right caudate nucleus on fluid-attenuated inversion recovery (FLAIR) images. Her complete blood cell count (CBC) showed an elevated white blood cell count of 17,600 cells/mm^3^, but no evidence of infectious disease was found. Cerebrospinal fluid (CSF) analysis indicated no increase in cell count, but slightly increased protein levels (50 mg/dL). She was suspected to have acute demyelinating encephalomyelitis and was administered 250 mg of phenytoin and 1000 mg of methylprednisolone. Early next morning, her headache had disappeared, walking had improved to normal, and oral prednisolone 30 mg/day was started. However, during a shower four days after the onset, she had a severe headache again. Brain MRI revealed new patchy lesions in the frontal and occipital lobes, then she was transferred to our hospital.

She had a history of taking aspirin for migraine and was being treated for iron deficiency anemia caused by menorrhagia at a nearby clinic. On the other hand, she had no complications that caused cardiovascular events such as hypertension. Her hemoglobin (Hb) level was 7.9 mg/dL one month before the onset, but 12.1 mg/dL just 2 days prior, showing a rapid improvement in her anemia. Her general physical findings were as follows: body temperature, 36.9 °C; blood pressure, 130/90 mmHg; pulse rate, 100 beats/min; no anemia or jaundice; and no notable findings on examination of the chest, abdomen, and whole-body joints and skin. At the time of transfer to our hospital, neurological findings revealed alertness, no meningeal signs, and no abnormalities in the central nervous system. In addition, her symptomology was negative for motor paralysis in her extremities, cerebellar ataxia, sensory impairment, and autonomic nervous system abnormalities. Laboratory findings are shown in Table 1. In her CBC, we observed an increase in leukocyte number, which was considered to be due to steroid administration, and normal hemoglobin level with microcytic hypopigmented changes, which suggested that her anemia was improving rapidly. Biochemical examination showed a slight increase in serum creatine kinase levels, which may have been associated with the seizures. In addition, the level of serum brain natriuretic peptide (BNP) was elevated, indicating cardiac dysfunction. No abnormalities were found in the biochemical test results, including autoantibodies suggestive of collagen diseases.

**Table 1:** Results of blood and cerebrospinal fluid analyses

	Value	Reference range
**Complete blood cell**		
White blood cell (/μL)	25,770	3,300 – 8,600
Neutrophil (%)	75.6	38.5 – 80.5
Lymphocyte (%)	17.2	16.5 – 49.5
Monocyte (%)	7.1	2 – 10
Red blood cell (/μL)	650 × 10^6^	386 – 492 × 10^6^
Hemoglobin (g/ dL)	15.5	11.6 – 14.8
Hematocrit (%)	49.1	35.1 – 44.4
MCV (fL)	75.5	83.6 – 98.2
MCH (pg)	23.8	27.5 – 33.2
MCHC (g/dL)	31.6	31.7 – 35.3
Platelet (/μL)	37.5 × 10^4^	158 – 348
**Biochemistry**		
Total protein (g/dL)	7.5	6.6 – 8.1
Lactate dehydrogenase (U/L)	248	124 – 222
Creatine kinase (IU/L)	620	41 – 153
Urea nitrogen (mg/dL)	11	8 – 20
Creatinine (mg/dL)	0.62	0.46 – 0.79
Ferrum (μg/dL)	65	40 – 188
UIBC (μg/dL)	283	180 – 270
TIBC (μg/dL)	348	246 – 410
Ferritin (ng/dL)	28	12 – 60
BNP (pg/mL)	509.7	0 – 18.4
Troponin I (ng/mL)	0.1415	0.01 – 0.39
**Serology**		
C-reactive protein (mg/dL)	0.08	0.00 – 0.14
Anti-nuclear Ab	< 40	< 40
Anti-DNA Ab (IU/ml)	< 2.0	< 6.0
CH-50 (U/ml)	51.4	30 – 50
PR3-ANCA (U/ml)	< 1.0	< 3.5
MPO-ANCA (U/ml)	< 1.0	< 3.5
Anti-ARS Ab	< 5.0	< 25.0
Anti-cardiolipin Ab (IgM)	< = 8	< 8
Anti-cardiolipin Ab (IgG)	< = 5	< 10
Lupus anticoagulant	1.09	< 1.16
**Cerebrospinal fluid**		
Appearance	watery and transparent	watery and transparent
Initial pressure (cmH_2_O)	25	50 – 180
Cell count (/μL)	3	0 – 5
Mononuclear cell (/μL)	1	
Polynuclear cell (/μL)	2	
Total protein (mg/dL)	57	10 – 40

*MCV* mean cell volume, *MCH* mean corpuscular hemoglobin, *MCHC* mean cell hemoglobin concentration, *UIBC* unsaturated iron binding capacity, *TIBC* total iron-binding capacity, *BNP* brain natriuretic protein, *Ab* antibody, *ARS* aminoacyl tRNA synthetase, *PR3-ANCA* serine protease 3-anti-neutrophil cytoplasmic, *MPO-ANCA* myeloperoxidase–anti-neutrophil cytoplasmic

**Fig. 1:**
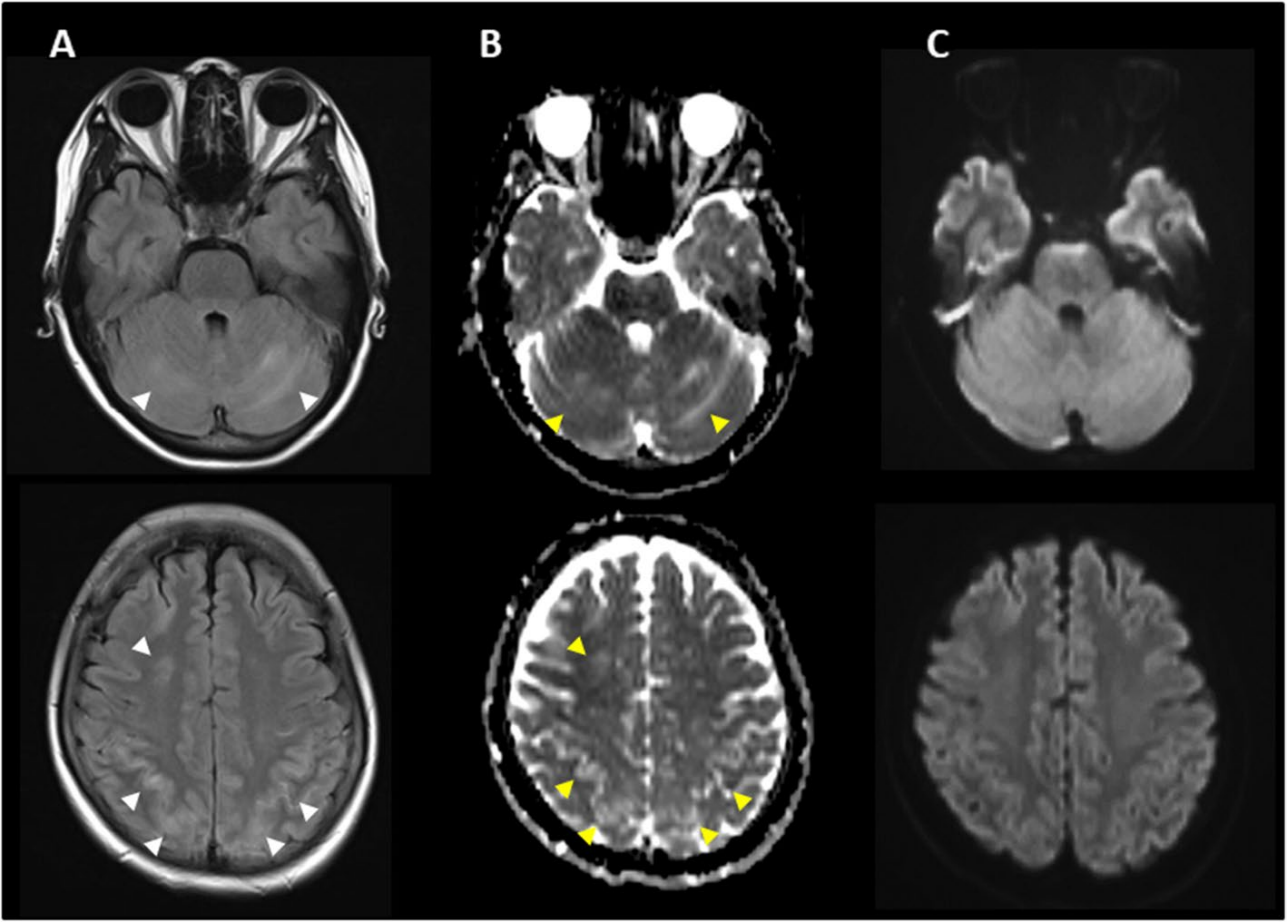
Brain magnetic resonance imaging (MRI) 5 days after onset of first headache. (**A**) Fluid-attenuated inversion-recovery (FLAIR) images showed hyperintense lesions in the right frontal lobe, bilateral parietal cortex and subcortex, and bilateral cerebellum (white arrow heads). (**B**) Apparent diffusion coefficient (ADC) map showed elevations in the same regions as the lesions observed (yellow arrow heads). (**C**) Diffusion-weighted image (DWI) showed no hyperintense regions

**Fig. 2:**
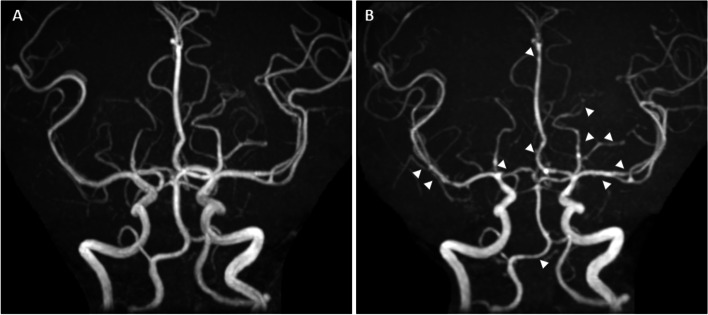
Brain magnetic resonance angiography (MRA) at onset of first headache and 4 days after the onset. (**A**) Brain magnetic resonance angiography (MRA) at onset revealed no abnormal findings. (**B**) Four days after the onset, vascular spasm with contraction and dilation of several main arteries with “string and beads” appearance was observed (white arrow heads)

**Fig. 3:**
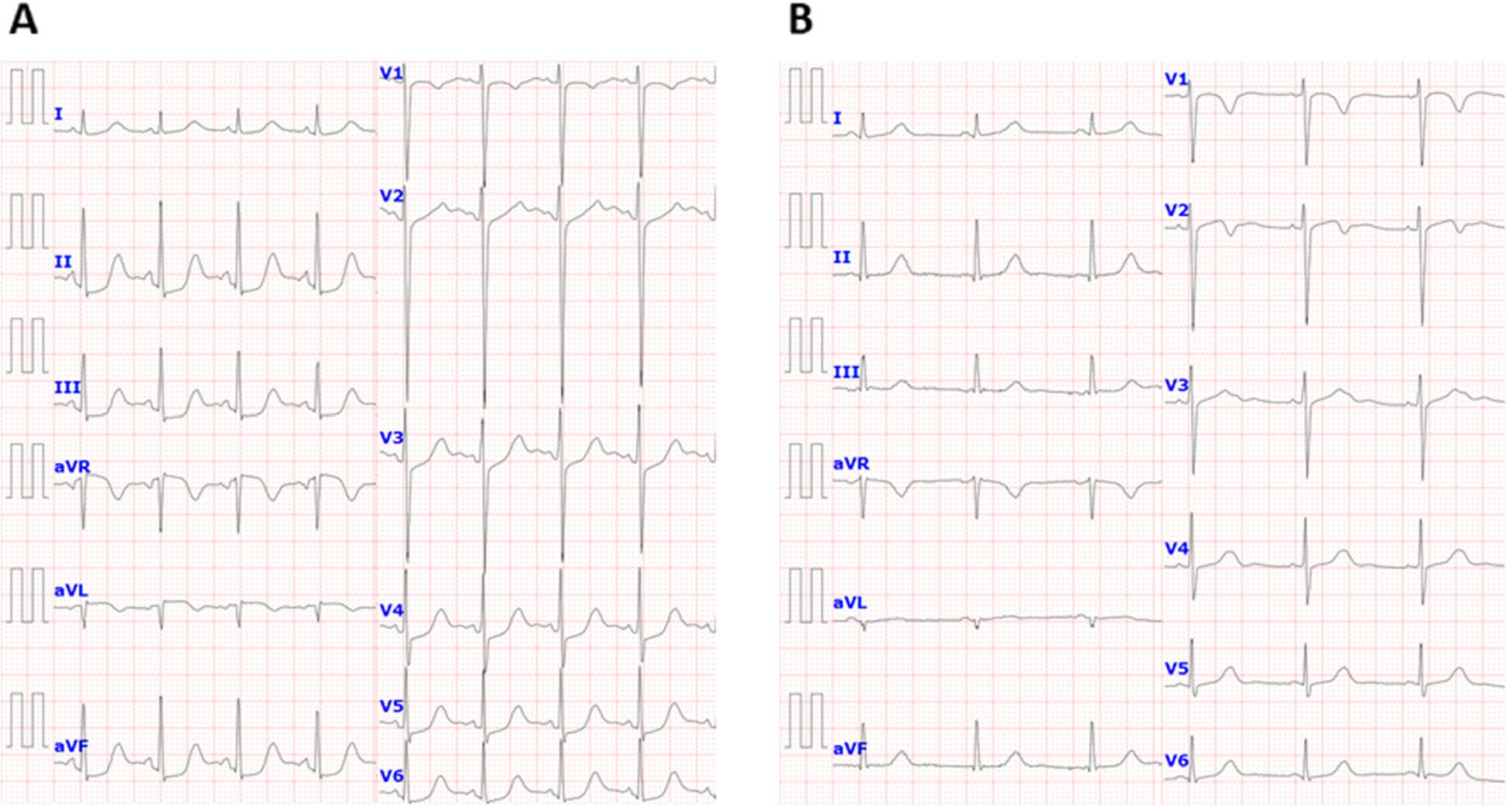
Electrocardiography (ECG) 5 days and 8 days after onset of first headache. (**A**) Electrocardiogram (ECG) 5 days after onset showed ST depression in limb leads II, III, and aV_F_, and in chest leads V_3_ to V_6_. (**B**) Eight days after onset, the changes were disappeared

**Fig. 4:**
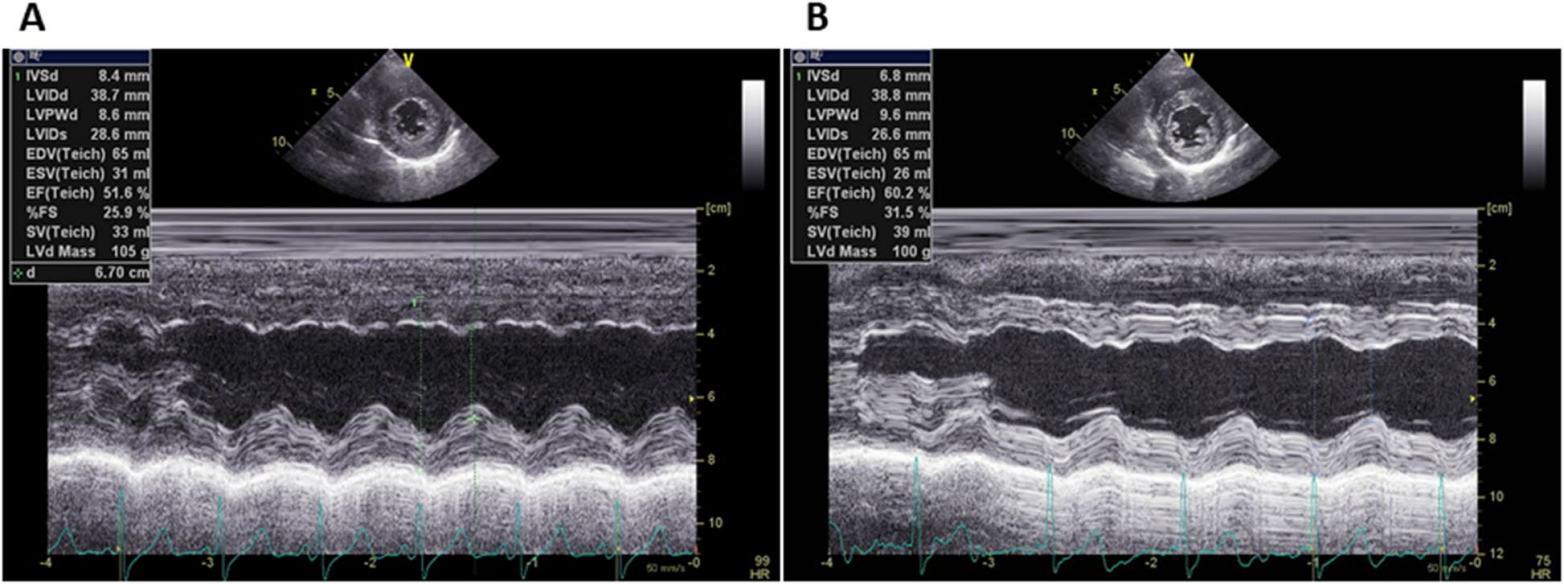
Ultrasound cardiography (UCG) 5 days and 8 days after onset of first headache. (**A**) Ultrasound cardiography (UCG) 5 days after onset revealed a decrease in wall motion on the left side at the base of the ventricular septum, with a slight decrease in ejection fraction (EF = 51.6%) and in fractional shortening (FS = 25.9%). (**B**) Eight days after onset, wall motion and EF/FS had improved

Five days after onset, brain MRI showed regions of hyperintensity in the right frontal lobe, bilateral parietal cortex and subcortex, and bilateral cerebellar lobes on FLAIR images (Fig. 1A). In addition, although the apparent diffusion coefficient (ADC) map showed elevations (Fig. 1B), no region of hyperintensity was observed on the diffusion-weighted image (DWI), suggesting angioedema (Fig. 1C). No abnormal findings were observed on brain magnetic resonance angiography (MRA) at the time of onset of the first headache (Fig. 2A). However, four days after the onset, multiple cerebrovascular spasms were observed, in which alternate contractions and dilations of several main arteries occurred (Fig. 2B). On the electrocardiogram (ECG) five days after the onset of the first headache, ST depression was observed in limb leads II, III, and aV_F_, and in the chest leads V_3_ to V_6_ (Fig. 3A). Ultrasound cardiography (UCG) showed a hypokinesis at the base of the side of intraventricular septum, with a slight decrease in ejection fraction (EF = 51.6%) and in fractional shortening (FS = 25.9%) (Fig. 4A). Although no symptoms of angina were observed 5 days after onset of first headache, left ventricular hypofunction leading to impending heart failure was suspected. Therefore, a calcium channel blocker and a nitrate were administered.

**Fig. 5:**
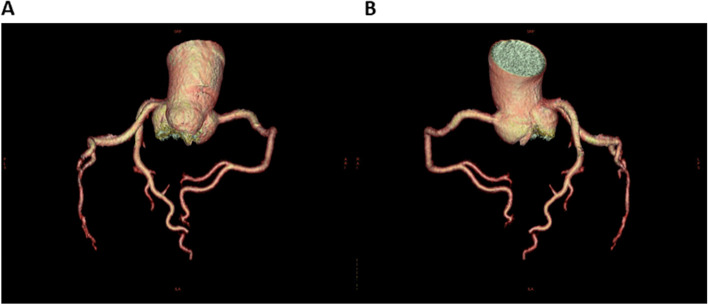
Computed tomography (CT) angiography at 7 months after onset of first headache. Coronary computed tomography (CT) angiography 7 months after onset showed no significant vasoconstriction in any coronary artery. Part (**A**) shows right anterior oblique (RAO) artery images, and part (**B**) shows left anterior oblique (LAO) artery images

Based on her clinical course and imaging findings, she was diagnosed with reversible cerebral vasoconstriction syndrome (RCVS) with cardiac involvement. We administered 5 mg of the calcium channel blocker amlodipine besylate, 25 mg of nitroglycerin, and 500 mg of the antiepileptic drug levetiracetam. The hyperintense regions on FLAIR images completely disappeared 23 days after onset of first headache. She showed no exacerbation of heart failure, and the ECG　(Fig. 3B) and UCG (Fig. 4B) findings were found to be normal eight days after onset of first headache. To avoid the risk of vascular spasm by iodinated contrast medium, we did not perform cardiac catheterization or coronary computed tomography (CT)-angiography until her RCVS was fully controlled. Since no recurrence of symptoms was observed after the treatment, we performed coronary CT-angiography seven months after the onset, which showed no significant vasoconstriction in any coronary artery (Fig. 5A, B).

## Discussion and Conclusion

The onset of symptoms in this case was with a thunderclap headache with mild weakness and convulsions in the left lower limb, and the severe headache recurred after bathing. Brain MRI revealed multiple subcortical white matter lesions in both the cerebellar hemispheres and cerebral hemispheres. Based on the diagnostic criteria [1], she was diagnosed to have RCVS. After oral administration of a calcium antagonist and nitrites, her severe headache and convulsions did not appear again, and her general condition gradually stabilized.

The triggers and background factors for RCVS include the postpartum period, migraine, hypertension, use of some drugs such as α-agonists, serotonin agonists, abuse of illicit drugs [2], rapid rise in Hb levels [3], and temperature differences such as bathing and showering with too cold or hot water [4]. In addition, it has been reported that RCVS worsens immediately after its onset by administration of steroids (which was in the history of our patient, as acute demyelinating encephalomyelitis had been suspected) [11]. Also, in our case, infections, vasculitis, and collagen diseases were excluded from clinical findings and blood test results. Thus, a history of migraine, improvement of anemia, showering, and post-onset steroid administration may have been the triggers and exacerbation factors in this case. It seems unique and instructive that the usual routine practice of prescribing iron supplements to anemic women was induced RVCS, although it is well known RVCS is caused after postoperative blood transfusion.

A notable feature of this case was that reversible cardiac involvement occurred concomitantly with RCVS. Despite the absence of chest symptoms, an increase in serum BNP levels, ST-T changes on ECG, and decreased EF and FS on UCG were seen and improved three days after therapy was started. This implies that there is cardiac involvement caused by Takotsubo cardiomyopathy or abnormalities in coronary arteries. So far, it has been reported that in a retrospective study 68 cases, from which 18 had patients had undergone UCGs around the period of active RCVS. Among them, three female patients who had no coronary artery disease or heart failure presented with wall motion abnormalities on UCG [9]. This report concluded that cardiac ventricular abnormalities may be a part of the RCVS spectrum. In another report, a middle-aged female patient with RCVS showed coronary artery spasm. The patient, with repetitive chest pain and transient wall-motion abnormalities on UCG, had experienced a thunderclap headache, and diffuse cerebral vasoconstriction was revealed on cerebral angiograms [10]; in this case, RCVS had developed after repeated coronary artery spasm. However, our case presented both cerebral and cardiac lesions almost coexisted. We did not perform the coronary angiography to avoid the adverse effect such as vasoconstriction by the contrast medium; however, coronary vasospasm may have occurred by the mechanism like that of cerebral vasospasm. Whilst, based on the UCG findings and ECG changes that cannot be explained by the distribution of coronary arteries, catecholamine cardiomyopathy such as reverse Takotsubo cardiomyopathy may have occurred by severe pain from thunderclap headache [11].

Our patient first visited a neurosurgery hospital for brain examination, but was eventually transferred to our general hospital because of recurrence of headache despite steroid administration. Although there were no chest symptoms at that time, cardiac involvement was confirmed as a result of cardiac evaluation, because BNP was abnormally high in the blood test at the time of transfer. Without a high level of suspicion by the clinician, RCVS may be underdiagnosed or the cardiac system may not be evaluated at all. To date, little attention has been paid to the involvement of extracerebral organs in patients with RCVS, but as reported in some cases, vasoconstriction may not be limited to the cerebral vasculature and may involve extracerebral organs. A cardiac involvement is sometimes a directly life-threatening issue, and should be recognized that both this and a cerebral lesion can coexist. Further accumulation of cases with RCVS should focus on identifying treatment options and defining the pathomechanism by which the cerebral and extracerebral lesions.

In this case, initial RCVS was triggered by a rapid improvement in anemia, and presented with transient abnormal cardiac function at the same time. RCVS diagnosis and therapy tend to be focused on neurological symptoms caused by cerebrovascular spasm, but it may be accompanied by decreased cardiac function.　Therefore, we should keep in mind the possibility of extracerebral involvement in RCVS.

## Data Availability

The datasets used and/or analyzed during the current study are available from the corresponding author on reasonable request.

## Abbreviations

ADC: apparent diffusion coefficient
BNP: brain natriuretic peptide
CBC: complete blood cell count
COVID-19: coronavirus disease 2019
CSF: cerebrospinal fluid
CT: computed tomography
DWI: diffusion weight imaging
ECG: electrocardiography
EF: ejection fraction
FLAIR: fluid-attenuated inversion recovery
FS: fractional shortening
Hb: hemoglobin
MRA: magnetic resonance angiography
MRI: magnetic resonance imaging
RCVS: Reversible cerebral vasoconstriction syndrome
UCG: ultrasound cardiography
